# Taphonomic signatures of early scavenging by black and turkey vultures

**DOI:** 10.1371/journal.pone.0307610

**Published:** 2024-08-14

**Authors:** Marian L. Wahl, Grant N. Burcham, Amanda M. Herbert, Lee A. Humberg, Patrick A. Zollner, Landon R. Jones, Brandon M. Quinby, Bryan M. Kluever

**Affiliations:** 1 Department of Forestry and Natural Resources, Purdue University, West Lafayette, Indiana, United States of America; 2 Heeke Animal Disease Diagnostic Laboratory, Dubois, Indiana, United States of America; 3 Department of Comparative Pathobiology, College of Veterinary Medicine, Purdue University, West Lafayette, Indiana, United States of America; 4 U.S. Department of Agriculture, Animal and Plant Health Inspection Service, Wildlife Services, West Lafayette, Indiana, United States of America; 5 Department of Wildlife, Fisheries and Aquaculture, Mississippi State University, Mississippi, United States of America; 6 U.S. Department of Agriculture, Animal and Plant Health Inspection Service, Wildlife Services, National Wildlife Research Center, Florida Field Station, Gainsville, Florida, United States of America; Universidad Austral de Chile, CHILE

## Abstract

Scavenging is critical for nutrient cycling and maintenance of healthy ecosystems. While there is substantial research into the identification of taphonomic signatures from facultative mammalian scavengers, early stage scavenging signatures by vultures remain unknown. Further, some vulture species are opportunistic predators, highlighting the need to define signatures observed in the course of normal scavenging behavior. We placed stillborn neonatal calves in an unoccupied pasture and used motion-trigger camera traps to quantify scavenging effort, then conducted necropsies to evaluate the effect of black vulture (*Coragyps atratus*) and turkey vulture (*Cathartes aura*) scavenging effort on carcass consumption. We measured the order of consumption of different tissue types to delineate which anatomic structures vultures consume first. Scavenging trials with higher numbers of vultures feeding on the carcass for longer were associated with decreased remaining tongue and abdominal viscera, and a larger umbilical wound. Greater maximum flock sizes were associated with decreased remaining tongue and abdominal viscera, a larger umbilical wound, and greater biomass consumption. Black vultures targeted the perineum and tongue earlier, while turkey vultures targeted the eyes, perineum, and tongue. These results are consistent with the idea that vultures prefer tissues that are easy to access and contain high nutrient content. These patterns form a distinctive taphonomic signature that can be used to identify early scavenging by black and turkey vultures. Our results demonstrate that criteria commonly used to identify livestock depredation by black vultures only document vulture presence and not predation. This distinction implies that new and more definitive criteria need to be developed and put into practice for more accurate decision criteria in livestock depredation compensation programs.

## Introduction

Scavenging plays a critical role in food webs [1]. A diverse assemblage of carnivores and detritovores consume carrion at varying rates, creating a gradient of scavenging species that range from obligate scavengers to varying levels of facultative scavengers [2, 3]. Within a food web, facultative scavenging may stabilize and increase the resilience of ecosystems by creating a complex system of trophic links between predators and detritus [3–5]. Further, both obligate and facultative scavengers provide a key role in nutrient cycling across many types of ecosystems [6, 7].

Among terrestrial vertebrates, vultures are the only obligate scavengers [8] and their scavenging provides vital ecosystem services by cleaning the environment of carrion [9]. Vulture scavenging, or the lack of it, impacts humans in multiple ways. Large mammal carcasses not scavenged by vultures decompose slower and attract mammalian carnivores [10] that can threaten livestock, thereby increasing the probablilty of human-wildlife conflict involving large carnivores. Catastrophic declines in vultures on the Indian subcontinent led to drastic increases in rat and feral dog populations and may lead to increased rates of diseases such as rabies and anthrax, which negatively affect human health and livelihoods [11–13]. Despite these compelling motivations, vulture scavenging behavior is poorly understood and understudied [14].

Among vulture species, a gradient between obligate and facultative scavenging also exists, with some species displaying predatory behavior [15, 16]. The details of scavenging behavior are crucial to understanding the predatory behavior of the black vulture (*Coragyps atratus*). Unlike the sympatric turkey vulture (*Cathartes aura*), black vultures are reported to attack vulnerable or newborn livestock, resulting in injury or death, especially to neonatal calves [17]. These losses are perceived to be widespread, with anywhere from 22–44% of livestock producers reporting losses to black vulture predation [18, 19]. The United States Department of Agriculture estimates that black vulture depredation results in the losses of 2,170 adult cattle and 24,600 calves in the US annually [20]. However, producers can overestimate losses to predators or misattribute losses due to other mortality sources, as was described for both griffon vultures (*Gyps fulvus*) [21, 22] and Andean condors (*Vultur gryphus*) [16, 23], as well as for mammalian predators [24–26]. As a species that occupies niches as both a scavenger and facultative predator, black vultures can transition between predation and scavenging. In situations in which black vulture predation of livestock is suspected, the details of scavenging sequence and timing are critical to determining if black vultures were the cause of mortality.

Characteristics and dynamics of scavenging are critical to understanding livestock depredation by wildlife [27]. For example, the presence of scavenging damage can obfuscate the ability to determine if livestock depredation occurred [28]. Specifically, understanding the sequence in which common scavengers consume different components of a carcass can provide insights into the presence of lesions as diagnostic of a predation event. Such insights into the sequence of scavenging can also provide information about relationships between the post-mortem interval and the detectability of co-morbidities. Vulture scavenging research has focused on measuring residual carcass components following the completion of scavenging [29, 30]. These studies assess features of late-stage scavenging, such as bone dispersal and skeletonization of pigs, rather than describing the early sequence of events during scavenging sessions of vultures on livestock carcasses [31, 32]. To date, one longitudinal study has been conducted to investigate the chronology of vulture scavenging of human remains, and focuses solely on late-stage events, such as body movement and bone dispersal [33, 34]. Further, past work on skeletonization suggests the presence of geographic variability in vulture scavenging patterns [35]. Together, these studies underline the need for detailed examination of feeding patterns across geographic locations and all stages of scavenging, particularly earlier stages of scavenging that may be useful for investigations of suspected livestock depredation [36].

Taphonomic signatures are the general patterns of scavenging damage exhibited by a species or taxonomic group and can be used to identify which species fed on a carcass. Elements of taphonomic signatures include the order in which tissues are damaged, characteristics of wounds, as well as patterns of disarticulation and scattering of body parts [37]. One widely applied diagnostic criterion is the identification of dental morphology from bite marks (reviewed in [38]). However, lack of dentition in birds makes this inadequate for identifying avian scavengers or distinguishing among species. While some signs of damage may be present on bones at late stages of scavenging, these lesions are faint and may be indistinguishable from bone modifications produced by carnivore teeth or from trampling of the carcass [31, 39]. Instead, habitual feeding behaviors and patterns of tissue consumption or scattering of carcasses play a greater role in vulture taphonomic signatures, characterized by targeting of soft tissues and extensive bone scattering [38]. As mentioned above, much of the published work regarding taphonomy of avian scavengers focuses on the sum total of scavenging activities over time, with emphasis on dispersal of skeletal remains. Recent work in Ontario characterizing seasonal effect on scavenger guilds briefly mentioned differences in avian scavenger taphonomy, with corvids (American crows and common ravens) and turkey vultures accessing tissue via “facial and rectal orifices” and bald eagles (*Haliaeetus leucocephalus*) tearing skin [40]. However, details concerning species preferences, order of target tissues, and characteristics of the wounds created by the birds were not measured.

Here, we aim to improve understanding of early scavenging effort by black and turkey vultures to inform wildlife managers, forensic investigators, and those interested in the ecology of scavenging. The first objective of this study was to measure and define the relationship between vulture scavenging effort and carcass state in animals known to have been consumed, but not predated by vultures. We hypothesized that the extent of carcass consumption is a function of scavenging effort. Accordingly, we predicted that measures of carcass consumption would increase with increased scavenging effort. Our second objective was to describe the sequence of tissue scavenged. We hypothesized that vultures would exhibit distinct consumption preferences for different tissue types. We predicted that vultures would consume tissues that are easy to access and nutrient-rich significantly earlier in the scavenging taphonomy.

## Methods and materials

To quantify vulture scavenging in relation to carcass state and determine scavenging sequence, we deployed calf carcasses for vulture consumption [41]. We collected calves that died <1 day after parturition, or were stillborn, from a commercial dairy operation. All carcasses were frozen immediately after death. Prior to use carcasses were thawed for a minimum of one day in a walk-in cooler at 1–2°C. All calves were deployed in the same position and location. Calves were not secured, as carcasses are too heavy to be moved by birds during early-stage scavenging. Calf body masses were collected before deployment. We deployed 20 carcasses in a laterally recumbent position on open ground in a pasture. This pasture was located at Purdue University’s Southern Indiana Purdue Agricultural Center (SIPAC) in Dubois County Indiana at 526896.53°E, 4255828.18°N. The carcass site was located roughly 50m from the nearest tree line, 300m from the nearest public road, and 10m from a gated two track road [41]. Deployments occurred between February and May, during the pasture’s recovery period between grazing events and consistent with the local spring calving season (Jason Tower, personal communication). The time between the arrival of the first vulture at a carcass and our retrieval of that carcasses averaged 96 minutes with a standard deviation of 90 minutes and a shortest period of exposure of 8 minute with a longest exposure of 377 minutes. No humans were present at the deployment site during data collection to minimize disturbance. Deployments occurred irregularly, at varying times of day ranging from 07:25 to 16:37, to reflect temporal variation in natural carcass availability. All data collection methods were approved by Purdue University’s Institutional Animal Care and Use Committee under protocol number 2004002035.

Carcasses were monitored using a live-feed game camera (Gocam Ghost Biological Edition, Spartan Camera, Johns Creek, GA, USA) that provided real time images of vultures present at the the carcass allowing us to monitor each deployed so we could control how long vultues scavenged it prior to retrival. After vulture exposure, calf carcasses were collected and necropsied. Time of collection was based on exposure estimates derived from deployment time and number of vultures present, and designed to encompass a range of scavenging levels. Necropsies were conducted by GNB, a board-certified veterinary pathologist, at Purdue’s Heeke Animal Disease Diagnostic Laboratory (ADDL), located on the SIPAC site. If carcasses were not immediately necropsied, they were stored in a walk-in cooler at 1–2° C for up to two days. A range of variables reflecting carcass state were measured during necropsies, including location, size, and shape of external tissue wounds and damage to soft tissues (Table 1). Remaining tongue length (mm) was measured from the rostral tip of the epiglottis. Abdominal viscera mass included the following, if present: gastrointestinal tissues, spleen, urinary bladder and associated structures, and tubular female reproductive tissues. Kidneys and other retroperitoneal structures were not included, as the narrow time window of scavenging and less accessible nature of retroperitoneal tissues led to no consumption of these structures during the study. The hindlimb musculature wound was calculated as volume of a rectangular prism, using a.) the dorsal-ventral measurement of the defect from the deepest extent of the wound to the level of the ischium and b.) two lateral measurements taken at the level of the greatest extent of the defect (typically, at the level of the ischium).

**Table 1:** Characteristics of stillborn calves measured in post-foraging necropsies. Calves were collected after varying levels of scavenging effort by black (*C*. *atratus*) and turkey vultures (*C*. *aura*).

Variable	Definition
Viscera ratio	Ratio of the post-scavenge mass of abdominal viscera remaining to the pre-scavenge calf mass.
Hindlimb muscle wound	Ratio of the volume of post-scavenge hindlimb muscle missing, calculated as a rectangular prism, to the pre-scavenge calf mass.
Perineal wound	Area of wound in the perineal region, calculated as an ellipsoid, in cm^2^.
Umbilical wound	Area of wound at the umbilicus, calculated as a circle, in cm^2^.
Biomass consumed	Ratio of the difference between pre- and post- scavenging masses to the pre-scavenging calf mass.
Tongue remaining	Length of tongue remaining after scavenging, measured from the base of the tongue, in cm.

We estimated total vulture scavenging effort using “vulture minutes” and maximum flock size. A motion-actived camera (Reconyx PC800 HyperFire Professional IR) was deployed 3m northeast of the carcass at a height of approximately 1.5m and angled slightly downwards towards the carcass to collect data on vulture scavenging activity. Photos were scored by eight observers, with one photo set scored by all observers to estimate potential observer bias. Observer bias was low, with >90% agreement in scores across observers. Within each photo we recorded the number of black and turkey vultures within one vulture length (~1 meter) of the carcass, representing animals that could potentially be actively feeding on the carcass. To estimate scavenging effort during each minute that vultures were present at the carcass we selected the image with the most vultures within one vulture length from the camera placed 3m from the carcass and counted that number of vultures. We then summed these values for each vulture species present from the time that carcass was deployed until it was retrieved. We used that sum as our estimate of vulture minutes associated with each carcass deployment. We measured maximum flock size as the greatest number of vultures of both species within frame during the duration of the calf deployment. We also used these photos to measure the order in which eye, tongue, perineum, umbilicus, and eponychium of the hooves was first damaged by each species of vulture. First damage was defined as first contact of a vulture beak with the tissue type.

We fit univariate regressions in R v4.0.5 [42] to model the effect of the independent variables (vulture minutes and maximum flock size) on the following dependent variables: tongue length, abdominal viscera remaining, hindlimb musculature wound, perineal wound, and umbilical wound (Table 1). Ratios using calf mass were used where possible to standardize data across calves of varying size. Vulture minutes and maximum flock size were highly correlated based on the Pearson correlation coefficient (0.72), and therefore were not included as predictors in models together. Linear, quadratic, and 3^rd^ degree polynomial models were fitted to each dependent/independent variable combination. Outliers were identified using Cook’s distance; observations were eliminated if they exceeded the 4/n threshold [43]. The most supported model for each analysis was selected using Akaike information criterion (AICc) in the package *AICcmodavg* [44]. No transformations were required to meet the assumptions of normality for any analysis. To assess if vultures consumed specific parts of the carcass earlier or later, we classified each of the five anatomic structures based on the sequence with which they were consumed during each carcass deployment using photos. The first and second tissues consumed during each experiment were classified as “early” while the fourth and fifth tissue consumed was classified as “late”. We created a 5x2 contingency table based upon structures and vulture species which we performed a Fischer’s exact test on, with p-values generated through Monte-Carlo simulations to adjust for multiple comparisons. Multiple pairwise comparisons were conducted to determine differences between each anatomic structure, using the R package *RVAideMemoire* [45]. Significant differences were determined as alpha ≤ 0.

## Results

**Fig 1:**
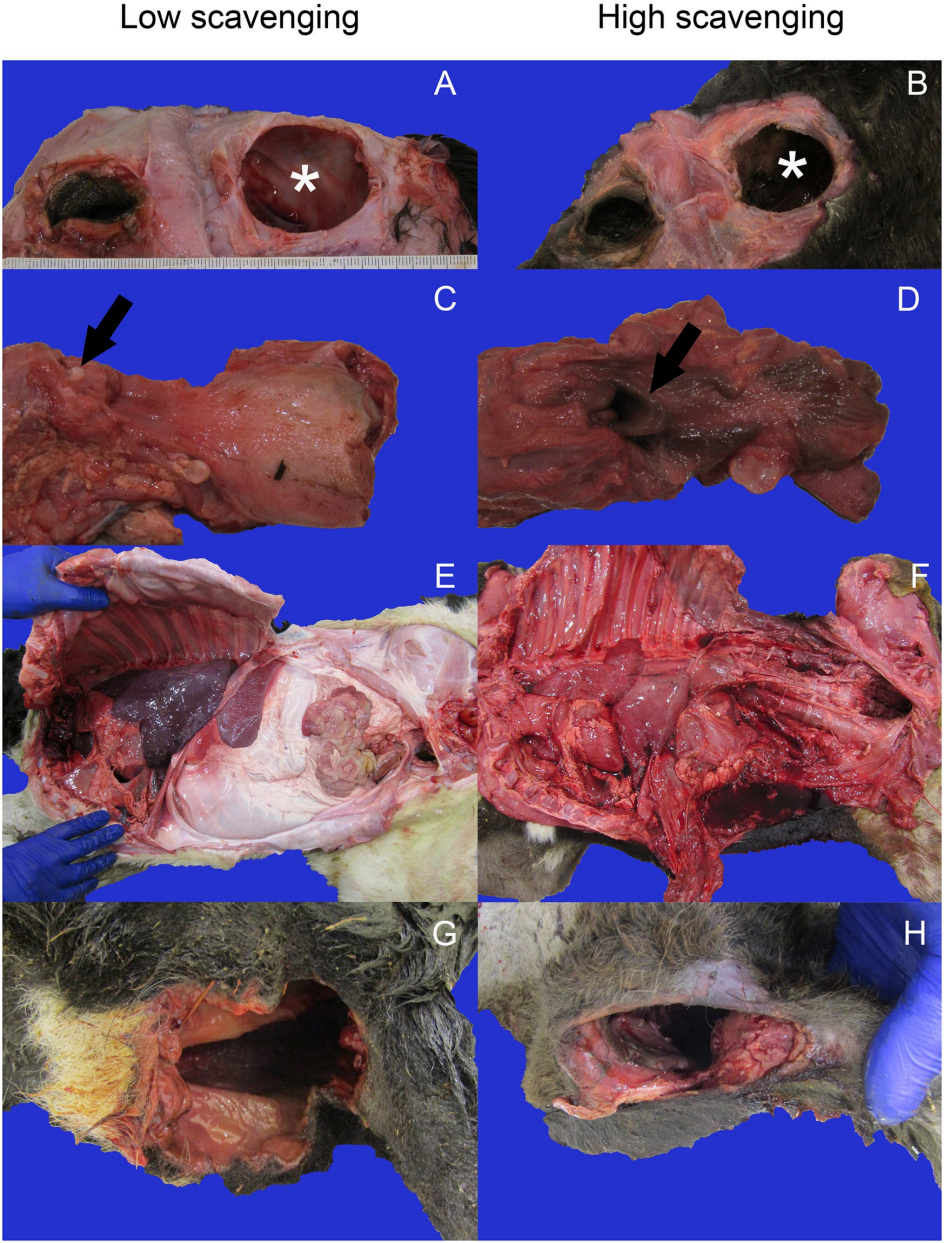
Characteristic patterns of changes in tissues observed in necropsies of stillborn calves scavenged by black (C. atratus) and turkey (C. aura) vultures. (A, B) Close view of the head of scavenged stillborn calves, concentrating on the orbit. Skin has been reflected cranially to reveal orbital subcutis. Asterisks denote the orbit. The globe is absent in situations of both low and high scavenging effort. Note lack of subcutaneous hemorrhage in orbital soft tissues. (C,D) Larynx, perilaryngeal tissues, and tongue in scavenged stillborn calves. Arrows denote the epiglottis. In low scavenging situations (C), the distal portion of the tongue remains. In higher scavenging situations (D), the entire length of the tongue is absent. In both situations, note lack of hemorrhage at proximal aspect of scavenged tissue. (E,F) Opened thorax and abdomen of scavenged stillborn calves; the head is to the left of the image. With low scavenging effort (E), abdominal viscera are intact. In contrast, high scavenging effort shows absence of most abdominal tissues. (G,H) Perineal region of scavenged calves. In both situations, a similarly-sized wound is present in the perineum, replacing the anus and vulva (if present). Note lack of hemorrhage in tissues at the edge of the wound.

Average duration of vulture exposure per carcass was 322 vulture minutes (SD = 517), with an average maximum flock of 6.1 birds (SD = 3.7) (Table 2). Flock composition ranged from 0% to 100% black vultures, with an average of 49%. The only observation of a species other than a black or turkey vulture during all of our data collection was a brief observation of a single red-tailed hawk (*Buteo jamaicensis*) during one deployment. At least one eye was absent on all calves but one (Fig 1) which had a relatively low total exposure time, at 85 vulture minutes. Vultures accessed the interior of the carcass via the perineum and the umbilicus. They did not create new wounds to access the interior of the carcass. While birds were occasionally observed pulling at skin on the sides and flank, a < 3 cm tear was only found on one calf, on the internal surface of the thigh, and no other cutaneous damage was observed. In one case, the left ear was removed by the aforementioned red-tailed hawk.

**Table 2:** Descriptive statististics of scavenging by black (*C*. *atratus*) and turkey vultures (*C*. *aura*) on stillborn calf carcasses. Vulture minutes reflects the sum of the total number of vultures present during the calf deployment. Maximum flock size reflects the largest number of vultures within one vulture length. Remaining variables reflect measurements of post-mortem gross lesions. SD represents standard deviation.

Variable	Mean	SD	Minimum	Maximum
Vulture minutes	322.46	517.86	8	2561
Maximum flock size	6.11	3.68	1	15
Viscera ratio	0.07	0.04	<0.001	0.12
Hindlimb wound (cm^2^)	54.26	82.54	0	344.73
Perineal wound (cm^2^)	29.31	21.79	3.06	87.18
Umbilical wound (cm^2^)	5.06	4.99	0	19.63
Biomass consumed	0.09	0.17	-0.49	0.39
Tongue (cm)	5.03	4.29	0	15

**Fig 2:**
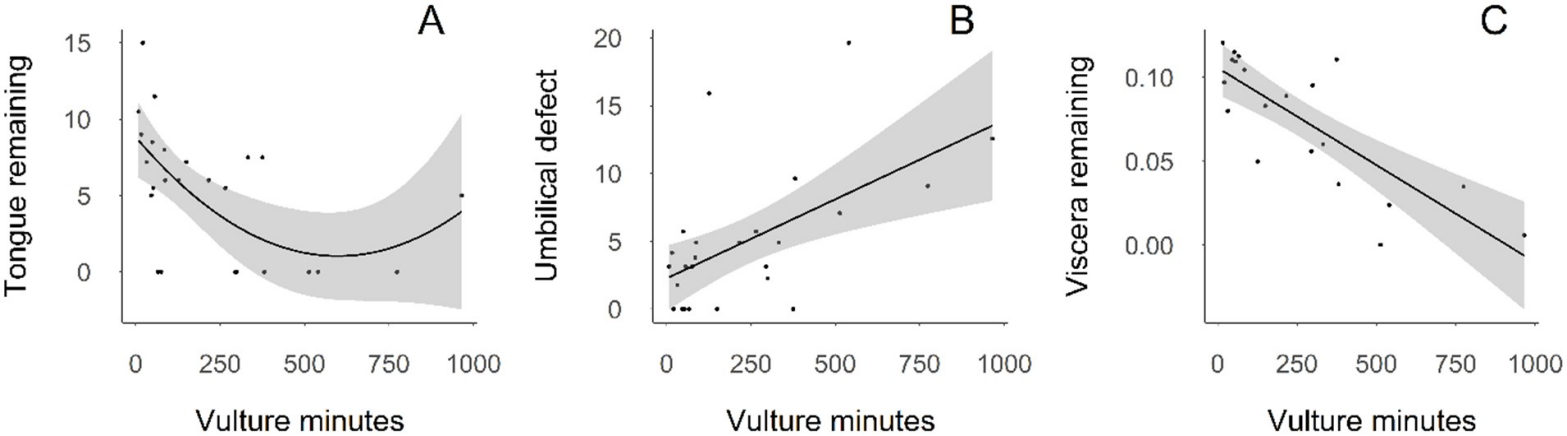
Relationship of three necropsy measurements with total scavenging exposure to black (*C*. *atratus*) and turkey vultures (*C*. *aura*), as measured by vulture minutes. Panel A shows length of calf tongue remaining as measured from the base of the tongue (cm), panel B shows the area of the calf umbilical wound (cm^2^), and panel C shows the remaining abdominal viscera, measured as the ratio of remaining viscera to pre-scavenge calf mass.

Remaining tongue length, umbilical wound area, and remaining abdominal viscera were significantly correlated with total scavenging effort, as measured by vulture minutes (Table 3a; Fig 1). Length of tongue remaining generally decreased with increased scavenging effort. However, our best model indicates that in rare instances with lots of vulture minutes observed (greater than 500 vulture minutes) then amount of tongue consumed was lower (Fig 2a). The area of the umbilical wound increased with increased scavenging effort (Fig 2b), while the mass of the remaining abdominal viscera decreased (Fig 2c). Abdominal viscera consumption began with tissues closest to the perineum, extending cranially with increasing vulture minutes. Vulture minutes did not significantly influence hindlimb muscle wound volume, perineal wound area, or total biomass consumed. While hindlimb muscle wound volume did not show a significant relationship with either of our predictor variables, the absence of hindlimb musculature adjacent to a relatively small perineal wound is part of the taphonomic signature we observed.

**Table 3:** Best fit models for each variable from linear, quadratic, and 3rd degree polynomial. Fit was based on AICc describing post-mortem characteristics of calf carcasses scavenging by black (C. atratus) and turkey vultures (C. aura). Characteristics are shown as a function of total vulture minutes (A) and maximum flock size (B). VM refers to vulture minutes and MF refers to maximum flock size. Beta refers to the regression coefficient.

**A**								
Variable	Type	df	F statistic	P value	Beta intercept (SD)	Beta VM (SD)	Beta VM2 (SD)	adj R2
Tongue remaining	quadratic	2,22	6.44	0.006	8.82 (1.24)	-0.03 (<0.001)	<0.001 (<0.001)	0.31
Umbilical wound	linear	1,23	11.3	0.002	2.27 (1.17)	0.01 (0.003)	-	0.3
Viscera remaining	linear	1,18	31.76	<0.001	0.12 (0.01)	<0.001 (<0.001)	-	0.62
**B**								
Variable	Type	df	F statistic	P value	Beta intercept (SD)	Beta MF (SD)	Beta MF2 (SD)	adj R2
Umbilical wound	linear	1,22	6.73	0.012	0.89 (1.54)	0.62 (0.24)	-	0.2
Viscera remaining	quadratic	2,16	15.9	<0.001	0.16 (0.02)	-0.02 (0.007)	<0.001 (<0.001)	0.62
Biomass consumed	linear	1,17	9.41	0.007	0.009 (0.03)	0.014 (0.005)	-	0.32

**Fig 3:**
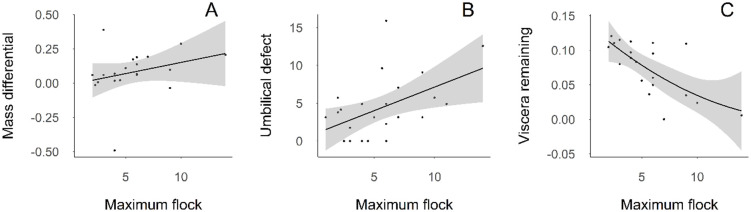
Relationship of necropsy measurements with maximum vulture flock size after scavenging exposure to black (*C*. *atratus*) and turkey vultures (*C*. *aura*). Panel A shows the total biomass consumed, panel B shows the area of the calf umbilical wound (cm^2^), and panel C shows the remaining abdominal viscera differential.

Umbilical wound area, and remaining abdominal viscera and weight differential were significantly correlated with maximum flock size (Table 3b). The difference in pre- and post- scavenging mass increased with larger maximum vulture flock size (Fig 3a), with post-scavenging calves weighing less. The size of the umbilical wound increased with larger maximum flock sizes (Fig 3b). Amount of abdominal viscera consumed increased with increased maximum flock size, tapering off at higher flock sizes (Fig 3c). Maximum flock size did not influence tongue remaining, hindlimb muscle wound volume, or perineal wound area.

Despite the fact that both vulture species consumed perineum and tounge earlier than other body parts, the order of body part consumption was significantly different between black and turkey vultures (p = 0.001 in each species). Specifically, pairwise comparisons demonstrated that black vultures consumed the perineum and tongue significantly earlier than other body parts, but no other comparisons were significant (Table 4). While similar pairwise comparisons revealed that turkey vultures consumed the eye, perineum, and tongue significantly earlier than the umbilicus or eponychium of the hooves (Table 4).

**Table 4:** P-values of pairwise comparisons for a contingency analysis for the order in which anatomic structures of stillborn calf carcasses were consumed earlier compared to later for black vultures (*C*. *atratus*) and turkey vultures (*C*. *aura*). Significant values are highlighted in bold. N = 20.

Black Vulture				
	Eye	Eponychium	Perineum	Tongue
Hoof	0.19	-	-	-
Perineum	0.07	**<0.001**	-	-
Tongue	0.54	**0.05**	0.22	-
Umbilicus	0.77	0.07	0.14	1
Turkey Vulture				
	Eye	Eponychium	Perineum	Tongue
Hoof	**0.02**	-	-	-
Perineum	1	**0.02**	-	-
Tongue	1	**0.02**	1	-
Umbilicus	**0.02**	1	**0.02**	**0.02**

## Discussion

Taphonomic signatures provide important clues to identify scavengers [38]. We found that carcass consumption by black and turkey vultures increased with time and flock size, supporting our first hypothesis that carcass consumption is a function of scavenging effort. We also found that black and turkey vultures exhibit a distinct taphonomy of tissue consumption, supporting our second hypothesis and prediction that vultures prefer easy to access tissues and tissues with high-nutrient concentrations.

Necropsy trends reinforced the importance of ease of access and nutritional concentration to taphonomic signatures from camera observations. Other New World vulture species, as well as other avian scavengers such as bald eagles, exhibit robust bills which are consistent with strong bite forces. Presumably, such bills permit them to tear open carcasses or enlarge wounds to access the majority of tissues for consumption [46]. In contrast, black and turkey vulture skull morphology suggests they have relatively little bite force, which would minimize ability to puncture the skin [47]. Such restricted abilities could limit these vulture species to consumption of exterior tissues or interior tissues through natural orifices, such as the perineum [46]. In our study, we found only one carcass with a small skin wound, supporting these conclusions.

Tradeoffs in ease of access based on vulture feeding limitations and nutrient content may explain the order of consumption and tissue preferences. Difficulty in accessing the umbilicus relative to the perineum or mouth may explain why vultures consumed this tissue later. Additionally, when cranially-located abdominal viscera become difficult to access as a vulture reaches limitations of how far its head and bill can reach through the pelvic canal, the umbilicus then provides a higher value access point. In contrast, the size of the perineal wound was not related to exposure time. This finding may be due to the comparatively thin skin of the perineum, which may be easier to tear, permitting the wound to become large early in the scavenging process. The presence of more substantial collagenous fascia in the subcutaneous space around the umbilicus may require more force to tear or open, thus, requiring more time and energy prior to scavenging this feature of the carcass. It is important to note that these patterns may change with calf age, as the umbilicus is completely healed within a few days after birth. In contrast, the volume of the hindlimb muscular wound and scavenging weight differential showed no significant relationship with exposure. While the hindlimb muscle has a relatively high ease of access through the perineal wound, viscera is composed of higher energy density tissues [48, 49], and is accessed through the same opening, with additional effort. This difference may lead to less predictable patterns of subsequent consumption of hindlimb muscle tissue. However, vulture scavenging should be considered as a possible cause when hindlimb musculature adjacent to a perineal wound is absent, without evidence of skin tearing. The finding that total biomass consumed was not influenced by vulture minutes may be driven by the fact that the high nutrient, easy access tissues that turkey and black vultures can access and consume make up a comparatively small amount of the total animal mass.

The sequence of tissue consumption supports the observed relationships with scavenging effort. Black vultures targeted the tongue and perineum first. Consumption of tongue tissue reflects high ease of access, and showed a clear correlation with scavenging effort. We did not expect the upward trend in remaining tongue length beyond 500 vulture minutes. This may represent increase competition between birds in larger aggregations leading to slowed consumption of the tounge. However, the confidence intervals in our modeled relationship were substantially wider at high values of vulture minutes; we would need to collect more data from calves that were deployed for very high values of vulture minutes to draw meaningful conclusions in that range of values. Similar to the tongue, the perineum is both easy to access and allows access to the high nutrient content abdominal viscera [48, 49]. The patterns we observed here are in line with previous reports, which often describe black and turkey vultures as targeting the eyes and tongue [14]. These results provide a foundation for further research on inter- and intra-specific interactions between scavenging birds [41] and differences between timing and patterns of scavenging.

Other life history and behavioral differences may explain why turkey vultures consumed eyes earlier than other body parts compared to black vultures. Turkey vultures have a strong sense of smell [50] and often arrive at carcasses before black vultures [51]. It may be that both species prefer consuming eyes but the typically earlier arrival of turkey vulture at carcasses affords them the opportunity to consume eyes before black vultures arrive. Although black vultures usually arrive later at carcasses, they often arrive in greater numbers and that might lead to them actively exclude turkey vultures from further consumption of carcasses. However, the details of the behavioral interactions of black and turkey vultures when scavenging carcasses remain poorly described [41].

Black and turkey vultures leave a substantially different scavenging taphonomy compared to many other co-occuring carnivores and other vulture species. Canid scavenging is characterized by tearing of skin and targeting high fat and muscle areas, with limbs targeted last ([52, 53], but see [54]). Bears similarly tear through skin, targeting abdominal viscera, upper limbs, and the axillary skeleton [38, 55]. Smaller scavengers display patterns more similar to our results. Corvids focus on eyes, tongue and soft tissue accessible through other wounds, and widen the margin of existing wounds [56, 57]. One hawk, the common buzzard (*Buteo buteo*), and the domestic chicken (*Gallus domesticus*) similarly target exposed soft tissue [38]. The Virginia opossum (*Didelphis virginiana*) targets viscera, accessed through the anus or existing abdominal tears, and scavenging is characterized by limited disturbance to the exterior of the carcass [58]. While rodents target existing wounds, rodent scavenging is also characterized by damage to lips, nose, and extremities [59]. Physical characterics of the scavenging species clearly influences ease of access to different portions of carcasses. In turn, the interaction of that access with nutrient composition impacts scavenging patterns.

Distinct patterns of scavenging can help identify scavenging species. In some species, such as bears and canids, the taphonomy is clear, with distinct diagnostic signs observable on skin, bone, and soft tissue [38]. In other species, such as corvids, hawks, and opossums, scavenging is difficult to distinguish [38]. Our results place vulture scavenging in line with the latter group, making close examination of the carcass in the early stages of scavenging critical to identifying vulture damage. Black and turkey vultures demonstrated highly similar tissue preferences. With black vulture predation on livestock becoming an increasing concern in the United States, identification of vulture damage and taphonomy can provide critical insights [17, 20, 60]. Our results show that characteristic patterns of vulture scavenging mimic those used by producers to identify predation events, including damage to tongue, eyes, and presence of perineal/umbilical wounds [19]. Further research should focus on determining if patterns of damage exist that could distinguish scavenging and predation events [36] and investigations comparing producer-reported livestock losses attributed to vultures with robust field studies that ascertain actual losses. Such previously described losses primarily rely upon qualitative or anecdotal accounts [61]. Our work fills in gaps concerning early vulture taphonomy on carcasses and is distinguished by our emphasis on quantifying relationships [36].

## Supporting information

S1 File Regression raw data. Raw data used in the regresission analysis reported in this paper.
(XLSX)

S2 File Contingency analysis raw data. Raw data used in the contingency analyis reported in this paper.
(XLSX)
